# Oncogenic enhancer of zeste homolog 2 is an actionable target in patients with non‐small cell lung cancer

**DOI:** 10.1002/cam4.1855

**Published:** 2019-08-27

**Authors:** Bin Shi, Carmen Behrens, Viralkumar Vaghani, Erick Marcelo Riquelme, Jaime Rodriguez‐Canales, Humam Kadara, Heather Lin, Jack Lee, Hui Liu, Ignacio Wistuba, George Simon

**Affiliations:** ^1^ University of Texas MD Anderson Cancer Center Houston Texas; ^2^ University of Texas School of Biomedical Informatics Houston Texas

**Keywords:** biomarkers, enhancer of zeste homolog 2, histone deacetylases, lung cancer, methyl transferase, non‐small cell lung cancer, prognosis, suberoylanilide hydroxamic acid, vorinostat

## Abstract

**Background:**

The aims of this study were to investigate the link between enhancer of zeste homolog 2 (EZH2) and histone deacetylase (HDAC) in preclinical studies and in human lung cancer tissue microarrays.

**Methods:**

Enhancer of zeste homolog 2 and HDAC1 mRNA expression in two lung adenocarcinoma (LUAD) datasets (MDACC and TCGA) were correlated with patient outcomes. We evaluated the association of EZH2 and HDAC1 expression with response to the HDAC1 inhibitor, suberoylanilide hydroxamic acid (SAHA). The response to SAHA was assessed at baseline and after alteration of EZH2 or HDAC mRNA expression in LUAD cell lines.

**Results:**

Direct correlation was found between EZH2 and HDAC1 expression (*P* < 0.0001). When EZH2 expression was knocked down‐ or upregulated, there was a corresponding decrease or increase in expression of HDAC expression, respectively. Cell lines with high EZH2 expression responded to SAHA treatment with a mean inhibition rate of 73.1% compared to 43.2% in cell lines with low EZH2 expression (*P* < 0.0001). This correlation was confirmed in non‐small cell lung cancer (NSCLC) specimens from MDACC (Spearman's correlation *r* = 0.416; *P* < 0.0001) and TCGA datasets (*r* = 0.221; *P* < 0.0001). Patients with high EZH2 and high HDAC1 expression in stage I NSCLC specimens of both datasets had the lowest survival compared to the patients with low expression of either or both markers.

**Conclusion:**

Our findings show that overexpression of EZH2 is a negative prognostic indicator. Increased EZH2 expression predicts for response to HDAC inhibitors and thus could serve as a biomarker for selecting NSCLC patients for treatment with HDAC inhibitors.

## Graphical abstract

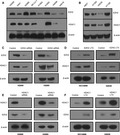

## 1 INTRODUCTION

Advances in our knowledge of the molecular pathways activated in lung cancer have led to the development of novel pathway‐directed targeted therapies. Epigenetic modulation of DNA and/or protein allows for the functional alteration of the proteome without structurally modifying the DNA.^1^ Methylation of lysine residues in histones is a key method by which chromatin and therefore gene function is modified.^2^

The methyltransferase enhancer of zeste homolog 2 (EZH2) is a central member of polycomb repressor 2 complex (PRC2), which plays a crucial role in the methylation of lysine 27 in histone 3 (H3K27). H3K27 is functionally activated in the methylated state.^3^ Enhancer of zeste homolog 2 is not only upregulated in several of the most common malignancies, including lung cancer, but also has been demonstrated to be a negative prognostic marker.^4^, ^5^

Enhancer of zeste homolog 2 combined with the embryonic ectoderm development (EED) protein serves as the catalytic subunit of the PRC2 complex and has been shown to interact with histone deacetylase (HDAC), another epigenetic modulator of histones. The EED/EZH2‐HDAC interaction has been shown to be highly specific, that is, HDAC does not interact with any of the other PRC2 protein complexes.^2^

It is now well known that acetylation is one of the common methods of epigenetic modification and is an important regulator of chromatin structure and function. Histone acetyltransferases acetylate the N‐terminus of the histone tails, leading to an open chromatin configuration which in turn facilitates gene transcription. Histone deacetylase, on the other hand, by deacetylating the N‐terminus of the histone tails, leads to a closed chromatin configuration and the transcriptional repression of genes. Histone deacetylase inhibitors have been shown to reverse the function of HDAC and thus induce growth arrest and apoptosis in non‐small cell lung cancer (NSCLC) cell lines.^2^, ^6^

Despite some initial promising early phase results, the development of HDAC inhibitors in lung cancer has been discontinued because of the lack of single‐agent efficacy and the lack of a biomarker that would facilitate patient selection.^7^, ^8^

We have previously described that increased expression of EZH2 in advanced NSCLC is a negative prognostic indicator and also a marker for chemotherapy resistance.^5^, ^9^ Given the interaction between EZH2 and HDAC, we evaluated the link between EZH2 expression and response to HDAC inhibition in NSCLC cell lines and explored the feasibility of using EZH2 expression as a biomarker of response to HDAC inhibitors in human tumors.

## 2 MATERIALS AND METHODS

### 2.1 Cell lines, cell cultures, and treatments

NSCLC cell lines were either originally purchased from the ATCC (Manassas, VA; H441 and H2009) or were obtained from Dr Adi Gazdar (The University of Texas Southwestern, Dallas, TX) (SK‐LU‐1, H1563, H4006, H2030, H1650, H4018, H827, H1355, H1195, and H2085). Cells were grown in RPMI 1640 high‐glucose medium supplemented with 10% fetal bovine serum (FBS) and maintained in a humidified 5% CO_2_ incubator. All cell lines used in the study were authenticated by short tandem repeat (STR) DNA fingerprinting using the PowerPlex 16 HS System (Promega, Madison, WI). Mycoplasma detection and eradication were accomplished using the MycoAlert™ plus mycoplasma detection kit and the MycoZap™ treatment kit, respectively (Lonza, Rockland, ME).

Four isogenic immortalized (BEAS‐2B and 1799), transformed (1198), and tumorigenic (1170‐I) cell lines were derived from in vitro lung carcinogenesis human bronchial epithelial (HBE) cells.^10^ These were a generous gift from Drs. Humam Kadara and Junya Fujimoto (The University of Texas MD Anderson Cancer Center, Houston, TX). The BEAS‐2B and 1799 cells were grown in keratinocyte serum‐free medium (K‐SFM; Gibco, Invitrogen Corp., Grand Island, NY), containing epidermal growth factor (5 ng/mL) and bovine pituitary extract (50 μg/mL). The 1198 and 1170‐I cells were maintained in K‐SFM media with 3% FBS (HyClone, Logan, UT) at 37°C in a humidified 5% CO_2_ incubator.

The four cell lines had been submitted for STR genotyping (Powerplex 1.2 at the Johns Hopkins CORE Fragment Analysis Facility, Baltimore, MD), and the results (obtained November 9, 2009) indicated that all four cell lines have an identical STR pattern, which was expected because the other three were derived from the BEAS‐2B cell line. The STR profile was as follows: AMEL: X, Y; CSF1PO: 9, 12; D13S317: 13, 13; D16S539: 12, 12; D5S818: 12, 13; D7S820: 10, 13; TH01: 7, 9.3; TPOX: 6, 11; vWA: 17, 18. This pattern was distinct from those of all other cell lines listed in the ATCC STR database of human cell lines in a search done on 16 November 2009.^11^

For most inhibition studies, lung cancer cells were treated for 4 days with the HDAC inhibitor suberoylanilide hydroxamic acid (SAHA, or vorinostat; Selleck Chemicals, Houston, TX) at a 2 µmol/L final concentration, the DNA methylation inhibitor 5‐aza‐2′‐deoxycytidine (AZA; Sigma, St. Louis, MO) at a 2 µmol/L final concentration, or the EZH2 inhibitor 3‐deazaneplanocin‐A (DZNep; Cayman Chemical, Ann Arbor, MI) at a 5 µmol/L final concentration.

### 2.2 Reagents

Suberoylanilide hydroxamic acid was dissolved at a concentration of 10 mmol/L in DMSO and diluted in growth medium before cell exposure. AZA was also dissolved in DMSO at a concentration of 10 mmol/L; aliquots were stored at −80°C. DZNep was dissolved at a concentration of 10 mmol/L in DMSO. It was diluted in growth medium before cell exposure. The stock solutions were diluted to the desired concentrations with culture medium before their use, keeping the final concentration of DMSO <0.1%. N‐nitrosamine 4‐(methylnitrosamino)‐1‐(3‐pyridyl)‐1‐butanone (NNK) and benzo[*a*]pyrene (Bap) were purchased from Sigma. It was dissolved at a concentration of 10 mmol/L in DMSO and diluted in growth medium before cell exposure.

In addition, 293 T cells were maintained in Dulbecco's modified Eagle medium (DMEM), supplemented with 10% FBS (15% FBS, Sigma‐Aldrich, St. Louis MO) 2 mmol/L glutamine, 100 U/mL penicillin, and 100 µg/mL streptomycin at 37°C in humidified air with 5% CO_2_.

Transfection of 293 T cells was performed using Lipofectamine 2000 (Invitrogen). Specific targeting of EZH2, HDAC 1, HDAC 3 in lung cancer cell lines was achieved by transient transfection with SMART pool‐designed siRNA (mixture of four different constructs). The siCONTROL nontargeting siRNA was used as a negative control (Dharmacon, Lafayette, CO). Transfection of the NSCLC cells by siRNA was performed using the siPORT™ amine transfection agent (Applied Biosystems, Carlsbad, CA) and Lipofectamine RNAiMAX reagent (Invitrogen). Transfection of the cells by plasmids was performed using Lipofectamine LTX (Invitrogen) as recommended by the manufacturer.

### 2.3 Antibodies

Antibodies obtained from Cell Signaling Technology (Danvers, MA) included HDAC1 (5356), HDAC2 (2545), and HDAC3 (2632). β‐Actin (A‐2228) was obtained from Sigma‐Aldrich The antibody SUZ12 (catalog number: sc‐46264) was purchased from Santa Cruz Biotechnology (Santa Cruz, CA). Anti‐EED (catalog number: 09‐774) and anti‐acetyl‐lysine (catalog number: 05‐515) were purchased from Millipore (Temecula, CA). The c‐Myc antibody (catalog number: 11667149001) was purchased from Roche Molecular Biochemicals (Indianapolis, IN).

### 2.4 Immunoprecipitation and Western blot analysis

Cell lysates were obtained using standard techniques. Lysis buffer contained 25 mmol/L HEPES (pH 7.7), 400 mmol/L NaCl, 1.5 mmol/L MgCl_2_, 2 mmol/L EDTA, 0.5% Triton X‐100, 0.1 mmol/L PMSF, 3 mmol/L DTT, phosphatase inhibitor cocktail (20 mmol/L β‐GP, 1 mmol/L Na_3_VO_4_; Roche), and protease inhibitor cocktail (10 µg/mL leupeptin, 2 µg/mL pepstatin, 50 µg/mL antipain, 1 × benzamidine, 2 µg/mL aprotinin, 20 µg/mL chymostatin; Roche). For immunoprecipitation, lysates were incubated with primary antibody overnight at 4°C. Agarose beads conjugated with A/G were then added and incubated for 2 hours at 4°C. The immunocomplexes were spun and washed three times with cold phosphate‐buffered saline (PBS) and once with lysis buffer. Immunocomplexes were then subjected to sodium dodecyl sulfate‐polyacrylamide gel electrophoresis (SDS‐PAGE). For Western blotting, 50‐80 µg of total proteins were electrophoresed on 6%‐12% SDS‐PAGE. The proteins were transferred to nitrocellulose membranes, probed with specific primary antibodies, and then probed with the appropriate horseradish peroxidase‐conjugated secondary antibodies (GE Healthcare, Waukesha, WI) Proteins were detected using a chemiluminescence‐based kit (GE Healthcare).

### 2.5 Methylthiazol tetrazolium assay

The cell proliferation rate was analyzed using a methylthiazol tetrazolium (MTT) assay. The colorimetric assay is based on the ability of live cells to reduce the MTT reagent (Promega) to a purple formazan product. The cells were seeded in 96‐well plates and treated with SAHA, AZA, and DZNep alone or together. A total of 100 µL of an MTT solution was added to each well, and the cells were then incubated at 37°C and 5% CO_2_ for 2 hours. After incubation, the cell viability was assessed by measuring the absorbance at 540 nm.

### 2.6 Total RNA isolation

Lung adenocarcinoma and normal lung tissue samples, obtained from resected lung cancer specimens from patients who had surgery at MD Anderson Cancer Center, were homogenized using Omni plastic disposable probes and an Omni (TH‐115) homogenizer (Omni International, Warrenton, VA) for 1 minute on dry ice. Total RNA was isolated from the cells using the Direct‐zol™ RNA Miniprep kit from Genesee Scientific (San Diego, CA) and samples homogenized using Trizol reagent according to the manufacturer's instructions. Total RNA was quantified using the Nanodrop 1000 spectrophotometer (Thermo Scientific, Wilmington, DE). RNA quality was assessed based on RNA integrity numbers generated by the Agilent Bioanalyzer 2000 (Agilent Technologies, Palo Alto, CA) according to the manufacturer's instructions.

### 2.7 Quantitative real‐time polymerase chain reaction

After isolation of total RNA, complementary DNA was synthesized by using the High Capacity RNA‐to‐cDNA RT‐PCR kit (Applied Biosystems, Foster City, CA). Quantitative real‐time polymerase chain reaction (qPCR) was performed by using the ABI 7300 fast RT‐PCR system (Foster City, CA). The conditions for the qPCR were 95°C for 10 minutes, followed by 40 cycles at 95°C for 15 seconds and 60°C for 1 minute. Primers to analyze the expression of HDAC1 (assay ID: Hs02621185_s1), HDAC2 (assay ID: Hs00231032_m1), HDAC3 (assay ID: Hs00187320_m1), EZH2 (assay ID: Hs00544833_m1), and GAPDH (assay ID: Hs02758991_g1; internal control) mRNA were purchased from Applied Biosystems.

### 2.8 EZH2 immunohistochemistry

The mouse monoclonal antibody against EZH2, NCLL (Novocastra, Leica Biosystem, Newcastle Upon Tyne, UK), was used at a 1:100 dilution. Immunohistochemical (IHC) staining was performed as previously reported using 5‐micron‐thick sections from tissue microarrays (TMAs). Tissue sections were deparaffinized and hydrated. Antigen retrieval was performed at a pH of 6.0 using a citrate buffer in a decloaking chamber (121°C for 30 seconds, 90°C for 10 seconds) and washed with Tris buffer. Peroxide blocking was performed at ambient temperature for 30 minutes with 3% hydrogen peroxide in methanol. Protein blocking was performed with Dako serum‐free protein (Agilent Technologies, Santa Clara, CA) block for 7 minutes. The slides were incubated with primary antibody at ambient temperature for 65 minutes and washed with Tris buffer, followed by incubation with Envision Dual‐Link System horseradish peroxidase (Dako) for 30 minutes. Staining was developed with 0.5% 3, 30‐diaminobenzidine, freshly prepared with imidazole‐HCl buffer, pH 7.5, containing hydrogen peroxide, and an antimicrobial agent (Dako) for 5 minutes. Slides were then counterstained with hematoxylin, dehydrated, and mounted. The nuclear immunostaining results for both markers were quantified jointly by two pathologists using a 4‐value intensity score (0, 1, 2, and 3) and the percentage (0%‐100%) of the extent of reactivity in each core. The final score was then obtained by multiplying the intensity and extension values (range, 0‐300) as previously reported.

## 3 PATIENTS

### 3.1 Patients with lung cancer

We analyzed mRNA expression data from two patient datasets:

1. In the first cohort, mRNA expression profile data from tumor tissues from 152 patients with primary lung adenocarcinomas who had undergone surgical resection between 1996 and 2009 at MD Anderson Cancer Center (MDACC) were included. This study was approved by MDACC's institutional review board. Clinicopathologic information was retrieved from the patient's electronic medical records. This dataset is henceforth referred to as the MDACC dataset.
2. In the second (validation) cohort, we analyzed The Cancer Genome Atlas (TCGA) lung adenocarcinoma database. This dataset is henceforth referred to as the TCGA dataset.

### 3.2 Statistical analysis

The chi‐square test or the Fisher exact test was used to test differences between discrete variables, and the Wilcoxon rank‐sum test or the Kruskal‐Wallis test was used to analyze the differences between continuous variable.^12^ Overall survival (OS) distributions were estimated using the Kaplan‐Meier method. The log‐rank test was used to determine survival differences between groups. Regression analyses of survival data based on the Cox proportional hazards model were conducted for OS. OS was defined as the time period from the time of diagnosis to death or last contact. Associations between protein expression and clinicopathologic variables were calculated using the median of the EZH2 expression as the cutoff.

SAS version 9.2 (Cary, NC) and S‐Plus version 8.04 (Palo Alto, CA) were used to carry out the computations. A *P* value (<0.05) was considered statistically significant.

## 4 RESULTS

**Figure 1:**
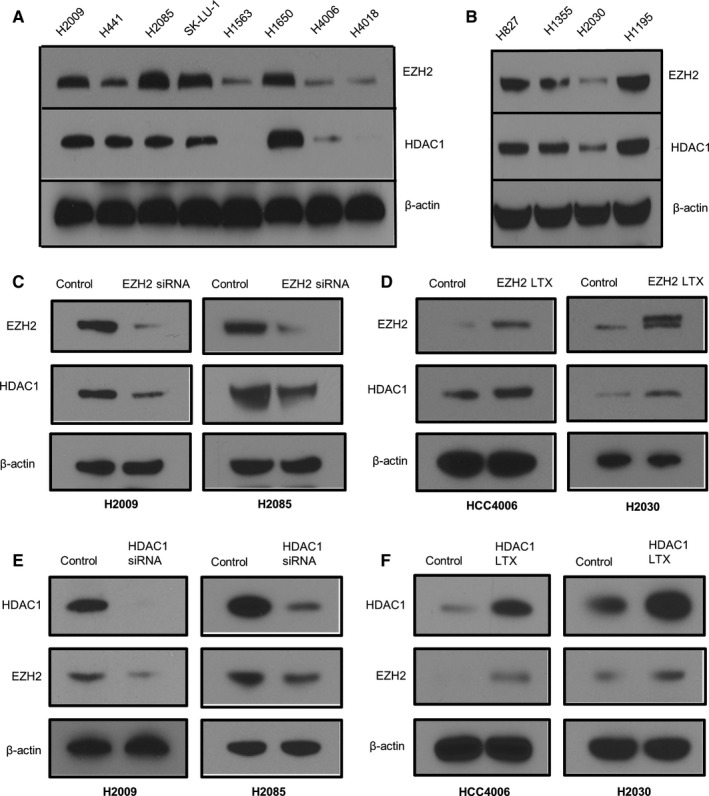
Enhancer of zeste homolog 2 (EZH2) and histone deacetylase (HDAC) expressions in non‐small cell lung cancer (NSCLC) cell lines are directly correlated. Figure panels A and B show Western Blots that demonstrate the direct correlation between EZH2 and HDAC expression in 12 NSCLC cell lines (8 cell lines with high EZH2 mRNA expression [H2009, H441, H827, H1195, H2085, SK‐LU‐1, H1650, and H1355] and 4 cell lines with low EZH2 expression [H4006, H4018, H1563, and H2030]). (A) and (B). HDAC1 protein expression decreases when EZH2 is knocked down in cell lines H2009 and H2085 (C). Increased HDAC1 protein expression is noted with forced EZH2 expression in cell lines H4006 and H2030 (D). Decreased EZH2 protein expression is noted when HDAC1 expression is knocked down in cell lines H2009 and H2085 (E). Increased EZH2 protein expression is noted when HDAC1 expression is forced in cell lines H4006 and H2030 (F)

We screened 74 NSCLC cell lines for EZH2 mRNA expression and selected 12 cell lines— eight with the highest and four with the lowest expression of EZH2—for further study. The eight cell lines highest in EZH2 mRNA expression were H2009, H441, H827, H1195, H2085, SK‐LU‐1, H1650, and H1355. The four cell lines lowest in EZH2 mRNA expression were H4006, H4018, H1563, and H2030. Please see Figure 1A,B.

### 4.1 EZH2 and HDAC expressions were correlated in NSCLC cell lines and in human NSCLC tumors

Enhancer of zeste homolog 2 and HDAC1 protein expressions were directly correlated in all the cell lines tested (Figure 1A,B). We confirmed this direct and positive correlation between EZH2 and HDAC1 in human tissue specimens. Enhancer of zeste homolog 2 and HDAC1 mRNA expressions were directly correlated in both the MDACC and TCGA datasets (Spearman's correlation *r* = 0.416; *P* < 0.0001 and *r* = 0.221; *P* < 0.0001, respectively). High EZH2 protein expression by IHC correlated with smoking status (current and former smokers vs never smokers; *P* = 0.001) and larger tumor size (*P* = 0.04) in the MDACC dataset.

**Figure 2:**
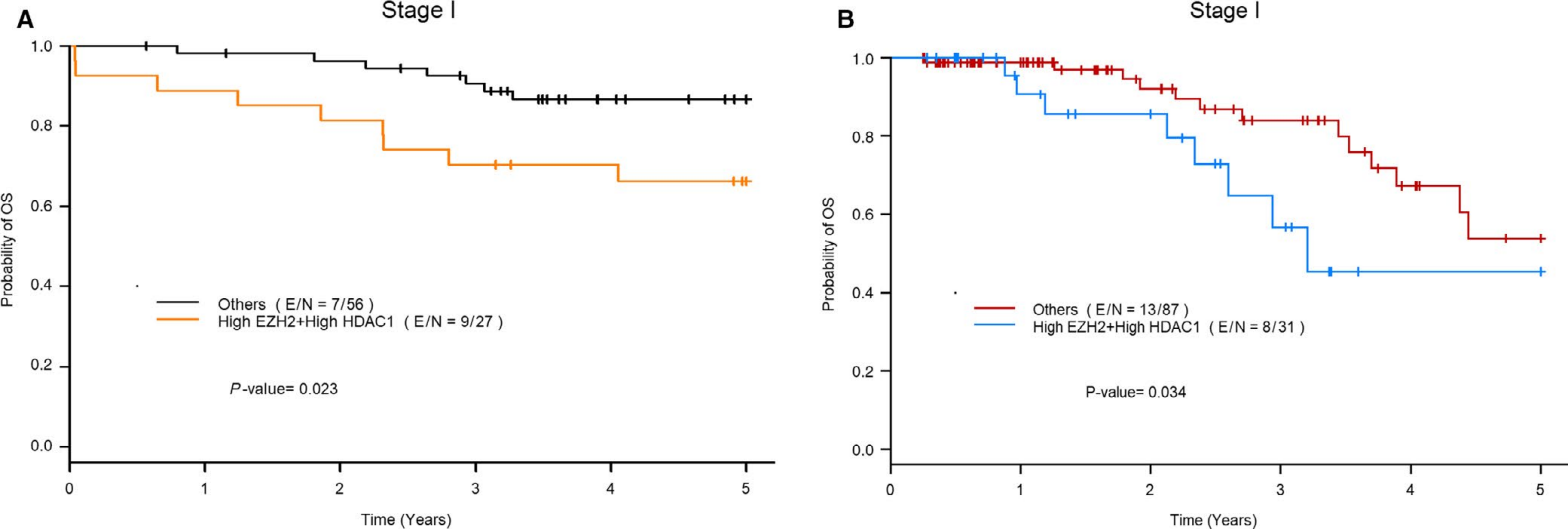
Overall survival of patients with stage I lung adenocarcinoma with high HDAC1 and enhancer of zeste homolog 2 expressions vs low expression of either or both markers (labeled as “others” in the graph) in the MDACC dataset (A) and the TCGA dataset (B)

Patients with stage I lung adenocarcinoma with both high EZH2 and high HDAC1 mRNA expression had worse OS as compared to patients with either or both markers low in both datasets (MDACC dataset: hazard ratio (HR = 2.97; *P* = 0.031 and TCGA dataset: HR = 2.6; *P* = 0.041) and multivariate analysis (MDACC: HR = 2.92; *P* = 0.034 and TCGA: HR = 3.17; *P* = 0.016; Figure 2). We also found a similar but nonsignificant trend for all stages in both the datasets.

### 4.2 Manipulation of expression levels of EZH2 leads to concordant changes in the expression levels of HDAC and vice versa

We then altered the expression of one molecule (HDAC1 or EZH2) and examined the effect on the other. When we knocked down EZH2 expression in the EZH2 over‐expressing cell lines H2009 and H2085, we noted a decrease in expression in HDAC1 at both the protein (Figure 1C) and mRNA (data not shown) levels. Reciprocally, the EZH2 mRNA and protein levels decreased after knocking down HDAC1 in the same two cell lines (Figure 1E).

To upregulate the expression of EZH2 and HDAC1, we transfected the EZH2 plasmid into the low‐EZH2‐expressing cell lines H4006 and H2030 and the HDAC1 plasmid into the low‐HDAC‐expressing cell line H4006. As shown in Figure 1D,F, forced expression of EZH2 not only led to an overexpression of EZH2 but also led to an increase in the expression of HDAC1. Likewise, forced expression of HDAC1 led to increased expression of EZH2. Thus, EZH2 and HDAC1 expressions are strongly correlated, and altering the expression of one leads to a corresponding alteration in the expression of the other.

### 4.3 High EZH2 expression predicts for increased sensitivity to HDAC inhibition

**Figure 3:**
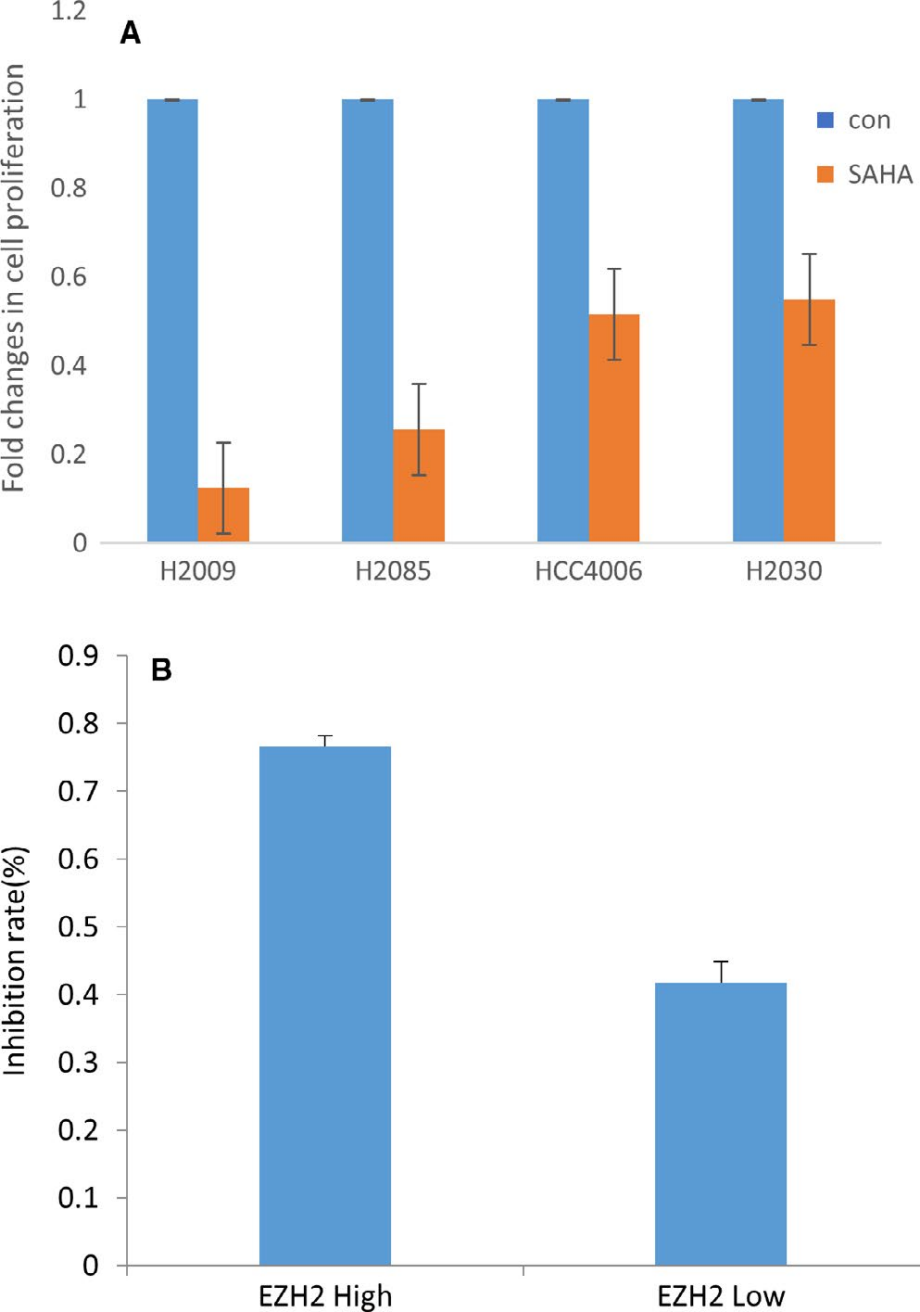
Enhancer of zeste homolog 2 (EZH2) expression and its sensitivity to histone deacetylase (HDAC) inhibition. Cell lines with high EZH2 expression (H2009 and H2085) demonstrate greater sensitivity to the HDAC inhibitor suberoylanilide hydroxamic acid (SAHA) than do cell lines with low EZH2 expression (H4006 and H2030), as measured by MTT assay (A). The differential response to SAHA treatment in high‐EZH2 vs low‐EZH2 cell lines taken in aggregate is shown here (B)

We then proceeded to test the effects of the HDAC inhibitor SAHA, the EZH2 inhibitor DZNep, and the DNA methyltransferase inhibitor AZA singly and in combination in two high EZH2 (H2085 and H2009) and two low EZH2 (H2030 and H4006) cell lines. MTT assays performed after treatment showed that all three drugs, alone and in combination, inhibited tumor cell proliferation. Significantly greater inhibition was seen with SAHA compared to the other two drugs (Figure 3). High‐EZH2‐expressing cell lines were particularly sensitive to the HDAC inhibitor SAHA. Additionally, the degree of response appeared to correlate with EZH2 protein expression as measured by Western blotting (Table 1). When the data from all 12 cell lines were considered in aggregate, the eight cell lines with high protein expression of EZH2 responded to SAHA treatment with an average inhibition rate of 73.1%, while the four cell lines with low EZH2 expression showed an average inhibition rate of 50% (*t* = 7.18 *P* < 0.0001; Table 1). Taken in aggregate, our data suggest that high EZH2 expression predicts for increased sensitivity to SAHA and could thus serve as a biomarker for increased sensitivity to HDAC inhibitors.

**Table 1:** Association between enhancer of zeste homolog 2 (EZH2) mRNA/protein, histone deacetylase (HDAC) mRNA/protein, and response to suberoylanilide hydroxamic acid (SAHA) in 12 non‐small cell lung cancer cell lines

Cell lines	SAHA inhibition (%)	EZH2 mRNA level	EZH2 protein level	HDAC1 mRNA level	HDAC1 protein level
H2009	87.6	High	High	High	High
H441	86.1	High	High	High	High
H2085	74.4	High	High	High	High
SK‐LU‐1	55.8	High	High	High	High
H1650	63.1	High	High	High	High
HCC827	77	High	High	High	High
H1355	73	High	High	High	High
HCC1195	68	High	High	High	High
H1563	70.2	Low	Low	Low	Low
HCC4006	48.4	Low	Low	Low	Low
HCC4018	36.4	Low	Low	Low	Low
H2030	45	Low	Low	Low	Low

### 4.4 Altering EZH2 levels alters sensitivity to the HDAC inhibitor SAHA

**Figure 4:**
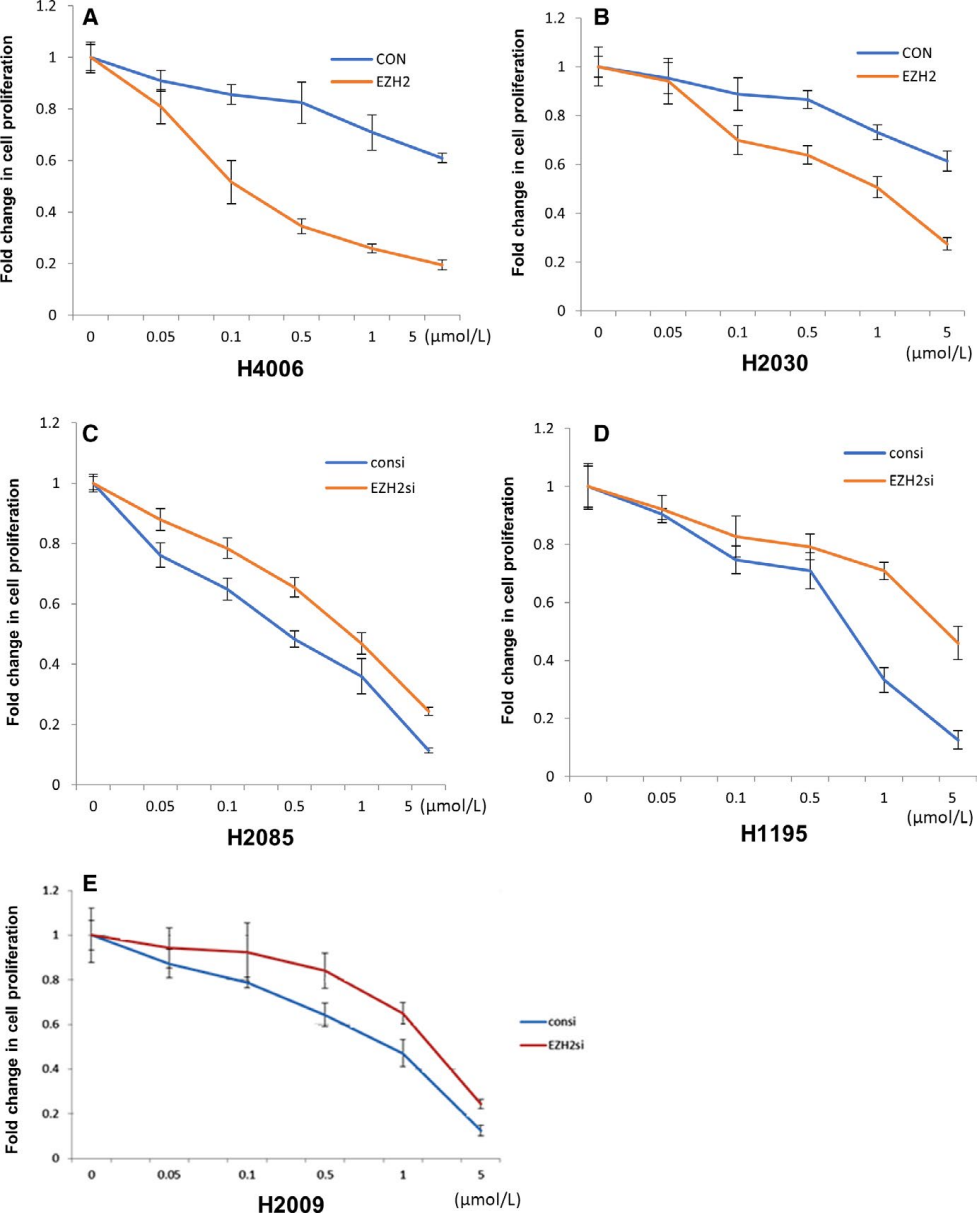
Altering enhancer of zeste homolog 2 (EZH2) expression alters the sensitivity to suberoylanilide hydroxamic acid (SAHA). Forced expression of EZH2 increases the sensitivity to SAHA in cell lines H4006 (A) and H2030 (B). Knockdown of EZH2 expression decreases the sensitivity to SAHA in cell lines H2085 (C), H1195 (D), and H2009 (E)

In order to confirm the correlation between EZH2 expression and SAHA treatment response, we knocked down EZH2 in two high‐EZH2 cell lines (H2009 and H2085) or overexpressed EZH2 in two low‐EZH2 cell lines (H2030 and H4006) to see if the sensitivity to SAHA would be correspondingly altered. MTT results showed that forced expression of EZH2 in low‐EZH2‐expressing cell lines led to increased sensitivity to SAHA (Figure 4). Similarly, the sensitivity to SAHA was decreased when EZH2 expression was knocked down. These data provide further evidence that high EZH2 expression predicts for increased sensitivity to SAHA and could thus serve as a biomarker of response to HDAC inhibitors.

### 4.5 Evidence for association between EZH2 and HDAC

**Figure 5:**
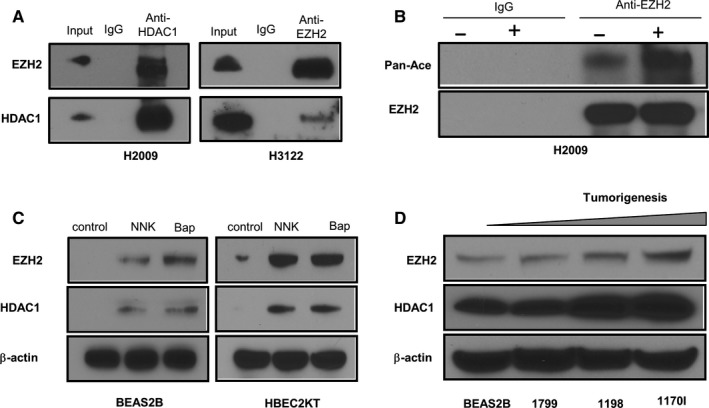
Evidence for association between enhancer of zeste homolog 2 (EZH2) and histone deacetylase (HDAC). The coimmunoprecipitation assay shows that EZH2 forms a complex with HDAC1 in the two lung cancer cell lines H2009 and H3122 (A). Treatment with suberoylanilide hydroxamic acid (SAHA) leads to pan‐acetylation (referred to as pan‐ace in the figure) of EZH2 (the + and − signs indicate with or without treatment with SAHA, respectively) (B). Western blot analyses show that both EZH2 and HDAC1 expressions increase with exposure to the smoking‐related chemicals N‐nitrosamine 4‐(methylnitrosamino)‐1‐(3‐pyridyl)‐1‐butanone (NNK) and benzo[a]pyrene (Bap) in human bronchial epithelial cell lines BEAS2B and HBEC2KT (C). The four isogenic premalignant and malignant cells lines BEAS2B (immortalized), 1799 (immortalized), 1198 (transformed), and 1170‐I (tumorigenic) show that EZH2 and HDAC1 expressions increase as the isogenic cell lines transitions toward malignancy (D)

We then sought to understand how EZH2 and HDAC are linked. The coimmunoprecipitation test confirmed that the two molecules are physically linked (data from H2009 and H3122 are shown in Figure 5A) and suggest that EZH2 and HDAC1 may form a complex. We further sought to discern whether EZH2 was post‐translationally modified by HDAC and whether treatment with SAHA would alter this post‐translational modification. As shown in Figure 5B*,* we found that acetylation of EZH2 is enhanced by inhibition of HDAC by SAHA. These data suggest that SAHA influences EZH2 function by its post‐translational acetylation.

To determine whether these genes could be coregulated, we screened the promoters of EZH2 and HDAC for common response elements (Table 2). Several transcriptional factors were found to bind the promoters of both genes, suggesting that these genes could be coregulated.

**Table 2:** List of common transcriptional factors with binding sites on the enhancer of zeste homolog 2 (EZH2) and HDAC1 promoters

Transcriptional factor (TF)	Number of binding sites transcription factor binding sites in the EZH2 promoter	Number of binding sites transcription factor binding sites in the HDAC1 promoter
AIRE	2	1
AML1	3	2
AP‐2a	2	2
BEN	4	2
Churchill	4	1
CPBP	9	12
Duxl	1	3
Egr‐1	1	2
HMGIY	1	2
HMX1	1	5
Homez	4	1
HOXB12	1	1
HOXC13	2	2
HOXD12	2	1
ING4	3	2
Muscle initiator	1	1
NF‐AT1	1	2
P53	1	1
Rhox1	1	4
Sp100	1	2
Smad4	1	1
Sox10	1	2
Sp1	2	1
SREBP	3	1
Zfp161	4	1
Zic1	1	2
ZNF333	1	5

We also sought to understand whether the expressions of these two molecules increase as tumorigenesis occurs and whether they remain correlated during this process. EZH2 and HDAC were evaluated in two different smoking‐related chemically induced lung cancer cell line tumor progression models. The first system comprised of four isogenic immortalized HBE cells transitioning from benign or parent to early premalignant to late premalignant and finally to malignant cells (immortalized BEAS‐2B and its derivatives 1799 [immortalized], 1198 [transformed], and 1170‐I [tumorigenic]). EZH2 and HDAC1 protein expressions increased with increasing malignant potential and remained correlated through the process of transformation (Figure 5D).

In a second set of experiments, we used the parent normal lung immortalized HBEC2KT cells induced to be tumorigenic by two tobacco‐related carcinogenic compounds: NNK and Bap. EZH2 and HDAC1 protein expressions increased with increasing malignant potential and remained correlated (Figure 5C). Furthermore, our cell line results are corroborated by our previously reported clinical data^13^ where we have shown that EZH2 expression both at the mRNA level and the protein level was correlated with smoking status with current or former smokers having higher expression levels compared to never smokers (*P* = 0.001).

## 5 DISCUSSION

High HDAC expression in several tumors including NSCLC predicts for a poor prognosis and suggests more aggressive tumor behavior.^14^ Several inhibitors of HDAC have been studied in NSCLC, including vorinostat (SAHA) and entinostat but have failed to improve survival in randomized studies.^7^, ^8^, ^15^ However, the NSCLC studies were done in an unselected patients. Our cell line and human data suggest that EZH2 could serve as a biomarker for the selection of patients who are likely to respond to HDAC inhibitors. Thus, randomized studies in advanced NSCLC patients selected on the basis of EZH2 expression are warranted.

We have previously demonstrated that EZH2 can be conveniently measured in formalin‐fixed and paraffin‐embedded human biopsy specimens.^5^, ^9^

Significantly, our findings also have chemo‐preventative implications. We show that the expressions of EZH2 and HDAC increase progressively from benign bronchial epithelial cells to lung cancer. We also show that these two proteins coimmunoprecipitate and that EZH2 function can be altered by its acetylation. The acetylation status of EZH2 could thus be influenced by the use of HDAC inhibitors. Others have previously reported that disturbances in epigenetic balance promote carcinogenesis.^3^ Overexpression of EZH2 has been implicated in cancer progression.^4^ Our findings therefore suggest that HDAC inhibitors could be used as a chemo‐preventative tool in patients of high‐risk patients.

In conclusion, our studies in human tissue microarrays demonstrate the negative prognostic significance of EZH2 and HDAC expression in patients NSCLC. Our cell line data suggest that high expression of EZH2 predicts for benefit with treatment with HDAC inhibitors. Further validation of these data in prospective clinical trials is warranted, where the efficacy of HDAC inhibitors is tested in patients with high EZH2 expression either as single agents or in combination with chemotherapy.

## CONFLICT OF INTEREST

The authors have no conflict of interest to declare pertaining to this work. The authors have not received any honoraria, served on advisory board(s), or served on boards of directors and do not hold stock in or receive royalties from the research companies mentioned in this manuscript.
